# SG-APSIC1088: Two-stage primary total knee arthroplasty for evolutive infected arthritis with coexistent joint destruction

**DOI:** 10.1017/ash.2023.30

**Published:** 2023-03-16

**Authors:** Wonchul Choi, Hyuk-Soo Han, Tae-Woo Kim, Moon Jong Chang, Joong Il Kim, Du Hyun Ro

**Affiliations:** CHA Bundang Medical Center, CHA University School of Medicine, Seongnam-si, Republic of Korea; Department of Orthopaedic Surgery, Seoul National University Hospital, Seoul National University College of Medicine, Seoul, Republic of Korea; Department of Orthopedic Surgery, Seoul National University Boramae Medical Center, Seoul National University College of Medicine, Seoul, Republic of Korea; Department of Orthopedic Surgery, Seoul National University Boramae Medical Center, Seoul National University College of Medicine, Seoul, Republic of Korea; Department of Orthopaedic Surgery, Hallym University Kangnam Sacred Heart Hospital, Seoul, Republic of Korea; Department of Orthopaedic Surgery, Seoul National University Hospital, Seoul National University College of Medicine, Seoul, Republic of Korea

## Abstract

**Objectives:** The treatment of infected knee arthritis in patients with coexisting joint destruction, including superimposed advanced arthritis or chronic osteomyelitis, is challenging. We investigated the outcomes of 2-stage primary total knee arthroplasty (TKA) for evolutive infected arthritis with coexistent joint destruction. **Methods:** We retrospectively reviewed the cases of 50 patients from 5 hospitals who presented with infected arthritis of the knee and were treated with 2-stage TKA: debridement and antibiotic-loaded articulating cement spacer (ALCS) insertion as the first stage and TKA as the second stage. We recorded demographics, laboratory results, and radiographic findings including Kellgren-Lawrence classification (KL) for knee arthritis. Outcomes including infection eradication, knee range of motion (ROM), and patient-reported outcome measures were evaluated. **Results:** The patient cohort had a mean age of 71.8 years (range, 40–86); they were followed for an average of 4.1 years (range, 2.2–13.3). Also, 40 patients showed KL grade 4, whereas 10 patients showed grade 3. A pathogen was identified in 38 cases (73.1%); methicillin-resistant staphylococci infections (N = 16) and *Candida* spp infections (N = 7) were the 2 most common types. Constrained prostheses were used in 10 cases (20%). Stem augmentations were used in 15 cases (36.0%) and block augmentations were used in 8 cases (19.0%). One patient had recurrent infection after TKA, so the eradication rate was 98%. After 2-stage TKA, the mean knee ROM was 119.4° (range, 80°–140°) and the mean Knee Society (KS) knee score was 90.4, the average KS function score was 84.7, the average Hospital for Special Surgery (HSS) score was 87.2, and the average Western Ontario and McMaster Universities Osteoarthritis Index (WOMAC) score was 16.7. The KS function scores (*P* = .029) and the WOMAC scores (*P* = .022) were significantly better in 17 patients who underwent ALCS insertion within 30 days of infection diagnosis compared to the other 33 patients. **Conclusions:** The 2-stage primary TKA for patients with infected knee arthritis with coexisting joint destruction showed satisfactory outcomes with a low infection recurrence. However, constrained prostheses or augmentation use may be necessary. Notably, some functional scores were better in the group that underwent ALCS insertion relatively early after the infection diagnosis.

